# JWA as a context-dependent redox regulatory hub linking stress responses with aging hallmarks and age-related diseases

**DOI:** 10.3389/fcell.2026.1981791

**Published:** 2026-10-29

**Authors:** Yaning Wu, Ning Zhang, Hui Sun, Heng Fang, Guangli Yan, Xijun Wang

**Affiliations:** 1 State Key Laboratory of Integration and Innovation of Classic Formula and Modern Chinese Medicine, National Chinmedomics Research Center, National TCM Key Laboratory of Serum Pharmacochemistry, Metabolomics Laboratory, Department of Pharmaceutical Analysis, Heilongjiang University of Chinese Medicine, Harbin, China; 2 State Key Laboratory of Quality Research in Chinese Medicine, Macau University of Science and Technology, Taipa, Macao SAR, China

**Keywords:** age-related diseases, aging, cellular senescence, DNA damage repair, JWA, oxidative stress, redox homeostasis

## Abstract

JWA (also known as ARL6IP5) is an evolutionarily conserved multifunctional protein increasingly implicated in redox homeostasis and cellular stress adaptation. Its functions intersect with redox homeostasis through signaling pathways including mitogen-activated protein kinase (MAPK), nuclear factor-erythroid 2-related factor 2 (Nrf2), and adenosine 5′-monophosphate-activated protein kinase (AMPK), as well as interactions with key proteins such as X-ray repair cross-complementing 1 (XRCC1), nuclear receptor coactivator 4 (NCOA4), and excitatory amino acid carrier 1 (EAAC1) involved in oxidative-stress adaptation. As a redox-responsive regulatory protein and mediator, JWA exhibits context-dependent roles in disease pathogenesis: in oncology, it acts as a tumor suppressor by mitigating oxidative stress-induced DNA damage and reversing chemoresistance, with reduced expression correlating with poor prognosis in gastric cancer, hepatocellular carcinoma, and other malignancies; in neurobiology, it exerts neuroprotective effects through suppression of ferroptosis, regulation of glutamate homeostasis, and proteostasis-related mechanisms, with the strength and direction of redox involvement varying across experimental contexts; beyond these fields, JWA regulates intestinal barrier integrity, radiation response, and adaptation to environmental oxidants (Cd, B [a]P, H_2_O_2_), while specific polymorphisms influence redox-related disease susceptibility. As a potential aging-associated regulator, accumulating evidence suggests that JWA expression or activity may change during aging, although age-, tissue-, and disease-specific patterns remain incompletely defined. Current evidence links JWA dysregulation to oxidative stress and tissue dysfunction in several age-associated diseases, whereas its direct roles in the aging process remain insufficiently established. This review, centered on redox regulation as the unifying framework, comprehensively synthesizes current knowledge on JWA’s multifunctional roles across disciplines, highlights its potential as a candidate redox-related biomarker and experimental therapeutic target, and proposes an evidence-based research framework for future investigation and translation. By emphasizing JWA’s role in maintaining redox balance and its regulatory function in aging process and age-related diseases, we provide novel insights into its significance in future clinical-development considerations and offer directions for targeting redox dysregulation in refractory diseases.

## 1 Introduction

JWA is an all-trans retinoic acid (ATRA)-responsive gene initially cloned from human tracheobronchial epithelial cells and maps to chromosome 3p14, a locus associated with cancer and epilepsy susceptibility (Wei et al., 2001). Evolutionarily highly conserved from *Caenorhabditis elegans* to humans, JWA encodes a cytoskeleton-associated protein that functions as a key mediator of redox homeostasis, linking oxidative stress signals to fundamental cellular processes (Wei et al., 2001; Butchbach et al., 2002; Wang et al., 2009). Unlike conventional redox regulators that act through single pathways, JWA can be viewed as a redox-responsive regulatory hub that integrates multiple stress-adaptation mechanisms, including modulation of redox-sensitive signaling cascades, regulation of DNA damage repair under oxidative stress, and participation in autophagic and proteostatic processes. Direct molecular sensing of ROS by JWA has not yet been established (Chen R. et al., 2007; Wang et al., 2009; Zhao et al., 2017).

The biological significance of JWA’s redox regulatory function spans diverse fields including oncology, neurobiology, environmental toxicology, and gerontology. In cancer, oxidative stress-induced DNA damage and redox imbalance drive tumorigenesis and chemoresistance, while JWA counteracts these processes by enhancing DNA repair and normalizing ROS levels (Wang et al., 2012; Xu et al., 2014). In neurodegenerative diseases, excessive oxidative stress and ferroptosis contribute to neuronal loss, and JWA exerts neuroprotection through ROS scavenging and inhibition of iron-dependent lipid peroxidation (Wang et al., 2018; Zhao et al., 2024). As an environmental stress-responsive gene, JWA is rapidly induced by oxidants such as H_2_O_2_, Cd, and B [a]P, indicating its responsiveness to cellular redox perturbation (Cao et al., 2007; Chen R. et al., 2007). As a potential aging-associated regulator, JWA expression has been reported to change with age, but the extent, tissue specificity, and functional significance of these age-related changes remain incompletely defined. Current evidence mainly links JWA dysregulation to oxidative stress and dysfunction in selected age-associated diseases rather than establishing a universal role in the aging process (Li et al., 2022).

Research on JWA has traditionally focused on its multifunctional roles in specific diseases, but the centrality of redox regulation as the unifying mechanism underlying its diverse functions has not been systematically elaborated. This review reorients the narrative around JWA’s role as a stress-responsive regulatory protein associated with redox homeostasis, integrating findings from tumor biology, neurobiology, and environmental toxicology to highlight its diverse roles in aging-related processes and age-associated diseases. We emphasize its dual potential as a candidate redox-related biomarker and experimental therapeutic target, propose a standardized research framework to address current methodological limitations, and outline an evidence-calibrated roadmap for future preclinical and clinical evaluation (Figure 1). By synthesizing current knowledge through the lens of redox regulation, this review provides a novel perspective on JWA’s biological significance and offers directions for future clinical-development considerations strategies targeting redox dysregulation in complex diseases.

**FIGURE 1:**
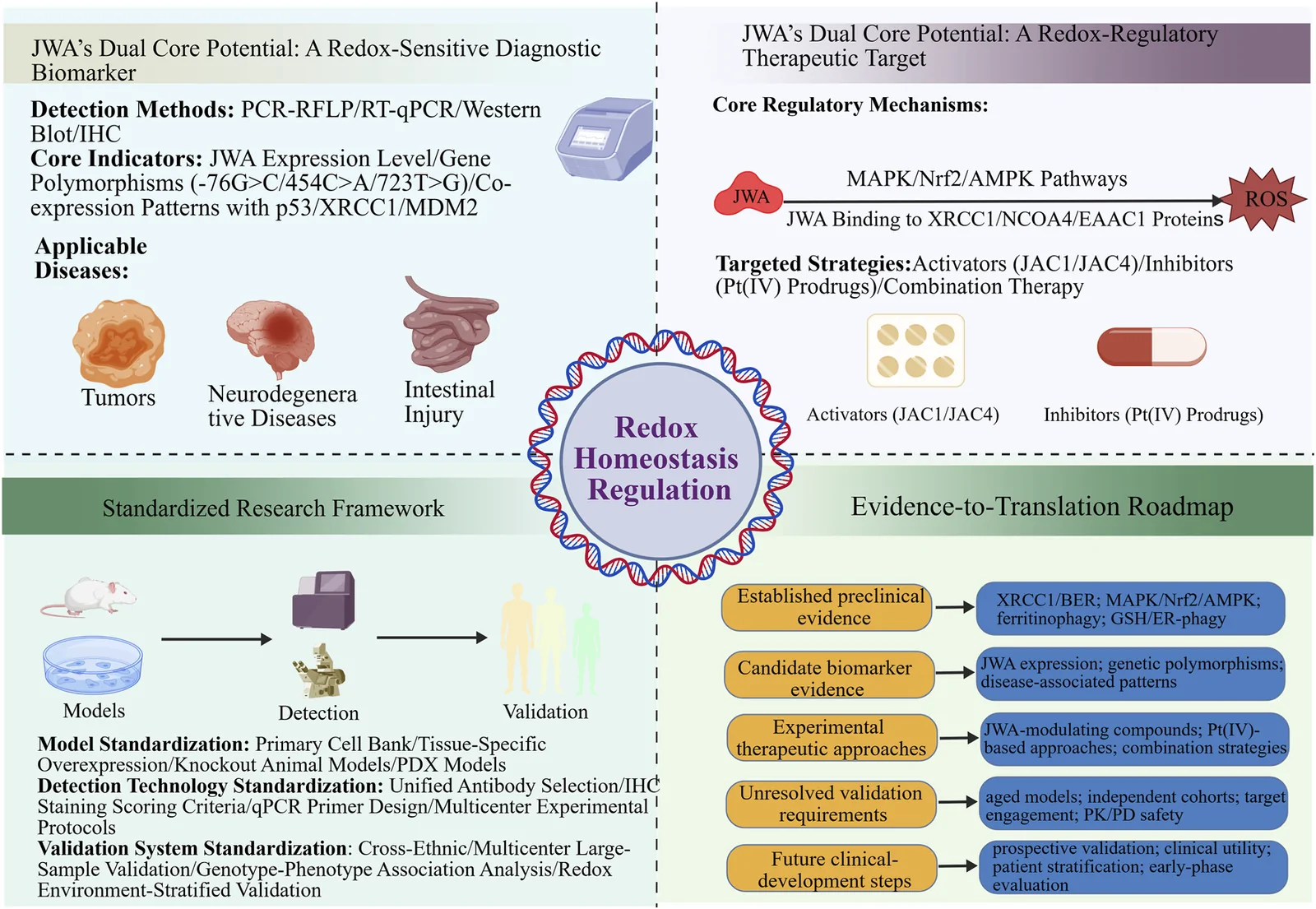
Evidence-to-translation roadmap for JWA research. Schematic overview illustrating the dual-core potential of JWA as both a candidate redox-related biomarker and a potential therapeutic candidate requiring further preclinical validation. Left section displays applicable disease categories and core detection readouts for JWA-based biomarker assays. Right section summarizes JWA-dependent core redox regulatory mechanisms and corresponding targeted intervention strategies (JAC1/JAC4 activators, Pt(IV) prodrugs, combination therapy). Bottom panels cover established mechanistic evidence, candidate biomarker findings, preclinical therapeutic approaches, unresolved validation requirements, and future clinical-development steps. PCR-RFLP, polymerase chain reaction-restriction fragment length polymorphism; RT-qPCR, reverse transcription quantitative polymerase chain reaction; IHC, immunohistochemistry; p53, tumor protein p53; JAC1, JWA gene-activating compound 1; JAC4, JWA gene-activating compound 4; Pt(IV), platinum (IV); PDX, Patient-Derived Xenograft. This figure was created using BioGDP (https://biogdp.com/zh).

This narrative review was developed through targeted searches of PubMed/MEDLINE, Web of Science, and Scopus, covering literature available through August 2026. Core search terms included combinations of “JWA,” “ARL6IP5,” “redox,” “oxidative stress,” “reactive oxygen species,” “aging,” “senescence,” “age-related disease,” “DNA repair,” “ferroptosis,” “autophagy,” “reticulophagy,” “biomarker,” and “therapeutic.” Additional studies were identified by screening the reference lists of relevant JWA-focused articles and recent reviews.

Priority was given to studies in which JWA was experimentally manipulated or directly measured and linked to a defined molecular mechanism and functional outcome. Generic studies addressing redox biology, aging, or disease pathways were used primarily for contextual interpretation and were not treated as direct evidence for JWA. Evidence was classified as (i) direct mechanistic evidence, when JWA perturbation was experimentally linked to a defined molecular event and functional outcome; (ii) context-dependent or disease-associated evidence, when JWA-related effects were demonstrated in a specific stress or disease model without establishing a universal causal relationship; (iii) candidate biomarker evidence, when JWA expression or genetic variation was associated with clinical or pathological features without prospective validation; and (iv) mechanistic extrapolation, when a biologically plausible relationship between JWA-associated pathways and aging hallmarks was proposed but not directly demonstrated.

## 2 Molecular biological basis of JWA-mediated redox regulation

### 2.1 Structural characteristics supporting redox function

The JWA gene maps to human chromosome 3p14, consisting of 3 exons, 2 introns, and a 567 bp intronless coding region that generates two transcripts (1447 bp and 2112 bp) (Wei et al., 2001; Butchbach et al., 2002). A key feature of its promoter is the enrichment of stress response element (SRE), heat shock response element (HRE), and retinoic acid response element (RARE), which enable rapid transcriptional activation in response to oxidative stress, ATRA, and 12-O-tetradecanoylphorbol-13-acetate (TPA) (Shen et al., 2007). Functional polymorphisms in the JWA promoter (-76G>C) and exons (380G>A, 454C>A, 723T>G) have been linked to altered redox stress sensitivity, increasing susceptibility to bladder cancer and leukemia by impairing JWA’s oxidative stress-induced transcriptional activation (Li et al., 2007; Shen et al., 2007).

JWA exhibits high interspecies homology, with the rat JWA coding region differing from humans by only 4 nucleotides (resulting in 4 amino acid changes that do not affect key functional domains) and mouse JWA (localized to chromosome 6D3) sharing a conserved gene structure (Wei et al., 2001; Butchbach et al., 2002). The JWA protein (≈21–22.5 kDa, 188 amino acids) is a hydrophobic, cytoskeleton-associated protein containing a conserved prenylated Rab acceptor 1 (PRA1) domain, transmembrane domains (TMDs), and kinase phosphorylation sites critical for redox signaling; The PRA1 domain, consisting of 4 TMDs, forms short hairpin structures via TMD1-TMD2 and TMD3-TMD4 pairing, which are essential for endoplasmic reticulum (ER) membrane shaping, oligomer formation, and ER-phagy regulation, key processes in mitigating ER stress-induced oxidative damage (Yamamoto and Sakisaka, 2025b; Yamamoto and Sakisaka, 2025a).

As a novel microtubule-associated protein (MAP), JWA stably binds α/β-tubulin regardless of microtubule polymerization status, localizing to the cytoplasm, microtubules, mitotic spindle, and partially to the ER/Golgi (Li et al., 2003). This localization enables JWA to integrate cytoskeletal dynamics with redox signaling, as microtubules serve as scaffolds for redox-sensitive kinases and ROS-producing enzymes (Chen H. et al., 2007; Bae et al., 2011). JWA’s redox regulatory functions are further supported by its direct interactions with key proteins: binding to NCOA4 to inhibit ferritinophagy and iron-dependent oxidative stress (Zhao et al., 2024), forming a complex with XRCC1 to enhance base excision repair (BER) of oxidative DNA damage (Wang et al., 2009), and associating with EAAC1 to regulate cysteine uptake and glutathione (GSH) synthesis, an essential antioxidant pathway (Lin et al., 2001; Watabe et al., 2007). Additionally, JWA contains two Protein Kinase C (PKC) phosphorylation sites (Serine-Aspartic acid-Arginine (SDR): 18–20 aa; Serine-Leucine-Arginine (SLR): 138–140 aa), which contribute to MAPK signaling activation under oxidative stress conditions and cellular adaptation to oxidative stress (Ikemoto et al., 2002; Chen H. et al., 2007; Qiu et al., 2018). The lysine 158 (K158) site is critical for ring finger protein 185 (RNF185)-mediated ubiquitination and degradation, and its mutation enhances JWA protein stability, potentiating redox protection (Qiu et al., 2018).

### 2.2 Redox-associated regulation of JWA expression

JWA expression is tightly regulated at transcriptional, post-translational, and extrinsic levels, with redox signals playing a central role in this regulatory network. This precise control ensures JWA can rapidly respond to redox perturbations and adapt cellular processes to maintain homeostasis.

#### 2.2.1 Transcriptional regulation by redox-sensitive factors

The JWA promoter contains multiple redox-responsive elements that mediate transcriptional activation or repression in response to oxidative stress. Nuclear Factor I (NFI), a multifunctional transcription factor involved in development and stress responses, specifically binds the CCAAT element (-58/-28 bp) of the JWA promoter to activate transcription under oxidative stress; NFI silencing abrogates this redox-induced upregulation (Chen R. et al., 2007; Tiberi et al., 2025). CCAAT/enhancer binding protein (C/EBP) family members, which regulate cell cycle and stress responses, bind the JWA promoter CCAAT box to modulate HeLa cell proliferation and apoptosis under oxidative stress conditions (Mao et al., 2006; Tolomeo and Grimaudo, 2020). The 12-O-tetradecanoylphorbol-13-acetate-response element (TRE) in the JWA promoter mediates TPA-induced transcription in a cell-specific manner (inhibiting in NIH3T3 cells, upregulating in K562 cells), linking JWA expression to redox-sensitive pathways involved in tumorigenesis and therapeutic sensitivity (Wei et al., 2001; Xu et al., 2025).

Transcriptional repressors also modulate JWA expression in response to redox signals. C-terminal binding protein 1 (CTBP1) directly represses JWA transcription, while Yin Yang 1 (YY1) binds the JWA promoter motif (5′-GAGAATGGCATA-3′) to inhibit expression, blunting JWA’s tumor-suppressive and pro-apoptotic effects under oxidative stress (Zhai et al., 2022; Ding et al., 2023b; Li et al., 2025; Tai et al., 2025). In contrast, inhibitor of growth family member 4 (ING4), a tumor suppressor that regulates chromatin structure, binds the JWA promoter (-107/-27 bp) to activate transcription, forming an ‘ING4-JWA’ axis that has been associated with reduced melanoma angiogenic signaling in experimental models, whereas whether redox normalization is a causal mediator of this effect remains unresolved (Lu et al., 2013a; Qu et al., 2025). The JWA -76C polymorphism reduces promoter transcriptional activity and abolishes oxidative stress-induced activation, highlighting the importance of promoter redox responsiveness in disease susceptibility and age-related redox imbalance (Li et al., 2007; Shen et al., 2007).

#### 2.2.2 Post-translational regulation: ubiquitination and phosphorylation in redox signaling

Ubiquitination and phosphorylation are key post-translational modifications that regulate JWA protein stability and stress-response functions. RNF185, an E3 ubiquitin ligase involved in protein homeostasis and stress responses, directly interacts with JWA, mediates its K158 site ubiquitination, and promotes proteasomal degradation, providing a mechanism for stress-associated regulation of JWA protein stability (Qiu et al., 2018; Liu et al., 2024). JWA also regulates the ubiquitination of downstream redox-sensitive targets: it facilitates ubiquitin-mediated degradation of transcription factor Specificity Protein 1 (Sp1) (Chen et al., 2014), which promotes cancer cell proliferation by upregulating tumor-associated genes under redox imbalance (Zhang et al., 2024; Hang et al., 2025). In normal cells, JWA maintains XRCC1 function to promote repair of oxidative stress-induced DNA single-strand breaks (Wang et al., 2009). In cisplatin-resistant gastric cancer cells, JWA inhibits casein kinase 2 (CK2) activity, blocking XRCC1 phosphorylation, promoting its ubiquitination/degradation, impairing DNA repair, and reversing cisplatin resistance; this links post-translational modification of JWA to oxidative-stress-associated chemoresistance (Xu et al., 2014; Chen et al., 2024; Lo et al., 2025). JWA’s PKC/Protein Kinase A (PKA) phosphorylation sites are critical for mediating stress-responsive MAPK signaling, as phosphorylation status directly affects JWA’s ability to regulate cell migration and oxidative stress responses, and its abnormal modification is associated with age-related cellular dysfunction (Chen H. et al., 2007). These apparently opposite effects of JWA on XRCC1 should not be interpreted as a single intrinsic direction of regulation. Instead, the available evidence suggests that the outcome is context-dependent and may be influenced by cellular state, basal redox environment, stress intensity, CK2 activity, XRCC1 phosphorylation status, DNA-repair demand, and proteasomal regulation. In normal cells exposed to oxidative stress, JWA is associated with preservation of XRCC1 function and BER capacity, whereas in cisplatin-resistant gastric cancer cells, JWA-mediated inhibition of CK2 and subsequent changes in XRCC1 phosphorylation and stability contribute to reduced repair capacity and drug resensitization. The molecular switch that determines these opposite outcomes remains unresolved and should be considered an important mechanistic boundary condition for future studies.

#### 2.2.3 Regulation by extrinsic redox modulators

Extrinsic factors, including differentiation inducers, environmental oxidants, and small molecules, regulate JWA expression in response to cellular stress and redox perturbation. ATRA, which induces cell differentiation and inhibits proliferation, enhances JWA transcription via the −194 to +107 bp promoter region; low-dose arsenic trioxide (As_2_O_3_) synergizes with ATRA to upregulate JWA, while TPA antagonizes this effect, thereby linking JWA to redox-sensitive differentiation pathways (Huang et al., 2006; Ami et al., 2026). Environmental oxidants and toxins (H_2_O_2_, B [a]P, Cd) upregulate JWA via oxidative stress signals or direct promoter binding, activating JWA’s redox protection functions, and this induction capacity declines with age, contributing to age-related oxidative damage accumulation (Cao et al., 2007; Chen R. et al., 2007). Small molecules and drugs modulate JWA expression to target redox imbalance: JAC1 upregulates JWA by promoting YY1 ubiquitination/degradation, inhibiting HSF1, blocking the cell cycle, and suppressing triple-negative breast cancer (TNBC) proliferation through the YY1-JWA-p38 MAPK pathway (Zhai et al., 2022). JAC4, a JWA-activating compound, exerts therapeutic effects on epidermal growth factor receptor (EGFR)-driven lung cancer growth and metastasis via the AMPK/Neural precursor cell expressed developmentally downregulated 4-like (NEDD4L)/EGFR pathway, the contribution of redox changes to this pathway remains unresolved (Ding et al., 2023b). Targeted agents (Cis-wog, Pt (IV) prodrugs) inhibit JWA-mediated redox signaling by binding JWA or interfering with its interaction with XRCC1 (Li et al., 2015; Ding et al., 2023a). Nicotine, whose metabolism generates ROS, negatively regulates JWA transcription via cholinergic receptor nicotinic alpha five subunit (CHRNA5)/protein kinase B (AKT), exacerbating redox stress and increasing disease risk (Feng et al., 2024; Craven et al., 2025; Gu et al., 2025; Teshima et al., 2026). Epigallocatechin gallate (EGCG), an antioxidant that scavenges ROS and promotes endogenous antioxidant enzymes, upregulates JWA via antioxidant effects, enhancing redox protection (Li et al., 2015; Ding et al., 2023a; Liu Q. et al., 2025; Shibata et al., 2025). JWA regulation exhibits cell specificity and pathological dependence: TPA’s effect varies by cell type; JWA is downregulated in Parkinson’s disease and aging (contributing to redox dysregulation), but compensatorily upregulated in prion-induced chronic ER stress to mitigate oxidative damage (Siddique et al., 2023; Kamble et al., 2025).

### 2.3 JWA-associated stress signaling pathways and cellular functions

In this review, “redox-dependent” is reserved for mechanisms in which experimental manipulation of redox status or ROS alters the JWA-associated pathway. Where such causal evidence is not established, we use “redox-sensitive,” “redox-responsive,” or “associated with oxidative stress” to describe the relevant biological context. JWA intersects with cellular stress and redox homeostasis through diverse signaling and protein-regulatory mechanisms, contributing to cellular adaptation and tissue homeostasis. Its functions are characterized by cell-type specificity and environmental dependence, with redox status representing either a mechanistic component or an experimental context depending on the pathway.

#### 2.3.1 JWA-associated tumor regulation in oxidative-stress contexts

JWA exerts tumor-suppressive effects through multiple pathways, including MAPK signaling, DNA damage repair, and target-protein degradation. Several of these mechanisms operate in oxidative-stress-associated tumor contexts, whereas direct causal dependence on redox status has been established only for selected processes (Wu X. et al., 2014; Xu et al., 2018). It forms a repair complex with XRCC1 to participate in the BER pathway, which repairs oxidative stress-induced DNA damage: low JWA expression enhances tumor cell sensitivity to platinum-based drugs by impairing BER, while JWA-targeted Pt(IV) prodrugs reverse chemoresistance by inhibiting the JWA-XRCC1 pathway, modulating oxidative-damage-associated DNA repair (Wang et al., 2012; Wang X. et al., 2020). JWA promotes proteasomal degradation of Sp1, thereby suppressing Sp1-associated phosphatidylinositol 3-kinase (PI3K)/AKT and nuclear factor-κB (NF-κB)/signal transducer and activator of transcription 3 (STAT3)/vascular endothelial growth factor (VEGF) tumor-promoting programs. These effects have been described in oxidative-stress-associated tumor settings, but direct causal dependence of JWA-mediated Sp1 regulation on redox status has not been established (Chen et al., 2014; Xu et al., 2018). Notably, the tumor-suppressive effect of JWA is weakened in elderly tumor patients, which is related to age-related JWA downregulation and redox imbalance (Chen R. et al., 2007; Li et al., 2022; Ding et al., 2023b).

JWA exhibits context-dependent functions in some cancers: in hepatitis C virus-associated hepatocellular carcinoma, it inhibits the MAPK/MMP-2 pathway in association with reduced oxidative-stress status, while accelerating tumor progression by promoting hepatitis C virus replication, highlighting the context dependence of JWA-associated signaling and the unresolved contribution of redox status (Hu et al., 2025). Co-expression patterns of JWA with p53 and XRCC1 accurately assess prognostic risk and chemotherapy benefits in gastric and esophageal cancer patients by reflecting the redox status of tumor cells, and this assessment value is more significant in elderly patients (Liu et al., 2012; Wei et al., 2015; Kim et al., 2024; Oren and Prives, 2024).

#### 2.3.2 Redox-mediated neuroprotection

JWA regulates neuronal homeostasis through context-dependent stress-response pathways, with protective effects demonstrated in several oxidative-stress and Parkinson’s disease-relevant models (Zhao et al., 2024). In Parkinson’s disease, JWA binds NCOA4 to suppress ferritinophagy, reducing iron accumulation and lipid peroxidation and thereby protecting dopaminergic neurons from ferroptotic injury (Zhao et al., 2024). It activates the Nrf2 pathway or upregulates glutamate transporter 1 (GLT-1) expression via the ERK/Akt/cAMP-response-element-binding protein (CREB) axis, enhancing glutamate uptake and alleviating excitotoxicity; both processes are redox-sensitive and critical for neuronal survival and preventing age-related neuronal loss (Zhao et al., 2017; Wang et al., 2018). JWA interacts with EAAC1 and regulates cysteine uptake and GSH synthesis, thereby contributing to cellular redox homeostasis and oxidative-stress defense (Lin et al., 2001; Watabe et al., 2007). In cognitive function regulation, JWA deficiency enhances hippocampal neurogenesis and long-term potentiation through the FAK/PI3K/AKT/mTOR pathway and improves spatial cognitive performance. Changes in redox balance have been associated with this phenotype, but a direct causal requirement for redox modulation in the FAK-dependent mechanism remains unresolved (Sha et al., 2016).

#### 2.3.3 Redox homeostasis and damage protection

JWA is involved in maintaining intestinal epithelial homeostasis, regulating oxidative stress responses, and providing radiation protection, with oxidative stress serving as an important biological context, and its dysfunction is an important cause of age-related intestinal barrier damage and radiation sensitivity increase (Li et al., 2022) via the ERK/F-box and WD repeat domain-containing (FBXW7)/notch homolog 1 (NOTCH1)/peroxisome proliferator-activated receptor γ (PPARγ)/signal transducer and activator of transcription 5 (STAT5) axis, JWA regulates intestinal stem cell function and cell lineage distribution, maintains intestinal barrier integrity, and promotes post-radiation injury repair by reducing redox stress (Li et al., 2022; Zhou et al., 2022). As an environmental stress-responsive gene, JWA is induced by H_2_O_2_, Cd, and B[a]P, alleviating DNA damage and inhibiting apoptosis by activating the BER pathway or regulating the ROS-MAPK signaling pathway, and this inducible protective capacity declines with age (Cao et al., 2007; Chen R. et al., 2007). As an ER membrane-shaping protein, JWA mediates ER tubule formation via the PRA1 domain and cooperates with family with sequence similarity 134, member B (FAM134B) to regulate ER-phagy, maintaining ER homeostasis and reducing ER stress-induced oxidative damage, which is critical for alleviating age-related proteostasis collapse (Pan et al., 2025; Yamamoto and Sakisaka, 2025b; Yamamoto and Sakisaka, 2025a). In prion infection, JWA induces reticulophagy/ER-phagy via the Ca^2+^-AMPK pathway, clearing abnormally folded prion protein (PRNP/PrP^c^) and alleviating ER stress, an important stress-associated mechanism, although the causal contribution of redox changes remains incompletely defined, and this mechanism also plays a role in clearing age-related abnormal protein aggregates (Kamble et al., 2025).

#### 2.3.4 Cell-specific and redox-context-dependent pathway characteristics

JWA’s downstream redox signaling pathways exhibit significant cell type specificity and environmental dependence, and also show age-dependent differences. In esophageal cancer cells (Eca109), it mainly regulates the ERK1/2 and c-Jun N-terminal kinase (JNK) pathways to maintain redox balance, whereas in normal esophageal cells (HET-1A), it relies solely on the p38 MAPK pathway (Lin et al., 2014). In normal gastric epithelial cells (GES-1), JWA positively regulates XRCC1 stability to enhance BER under oxidative stress; in cisplatin-resistant gastric cancer cells (BGC823/DDP, SGC7901/DDP), it promotes XRCC1 degradation to reverse chemoresistance by modulating oxidative-stress-associated DNA repair (Xu et al., 2014). In aging cells, JWA’s regulation of MAPK pathway subtypes is altered, leading to weakened redox balance maintenance capacity (Chen et al., 2026; Wang et al., 2018; Li et al., 2022). These differences highlight JWA’s ability to adapt its redox regulatory mechanisms to specific cellular contexts and aging status. The major JWA-centered mechanisms described above are integrated into a unified context-dependent regulatory framework (Figure 2).

**FIGURE 2:**
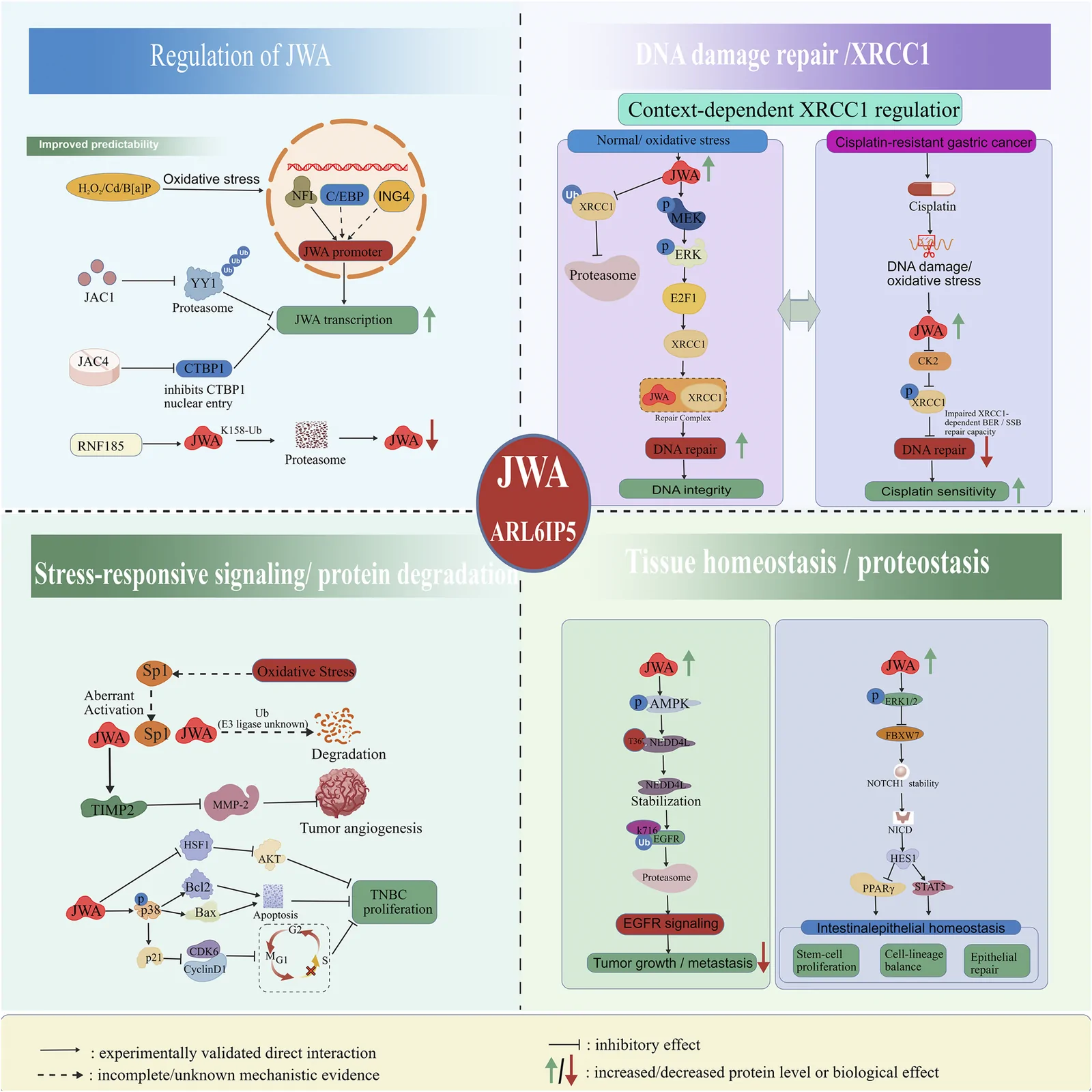
Integrated regulation and context-dependent effector programs of JWA. A unified cellular model integrating the major mechanisms regulating JWA expression, protein stability, DNA-damage responses, stress-responsive signaling, protein degradation, and tissue homeostasis. Extrinsic stressors and transcriptional regulators modulate JWA expression, whereas RNF185-mediated K158 ubiquitination contributes to JWA protein degradation. In oxidative-stress contexts, JWA interacts with XRCC1 and supports base-excision repair through MEK-ERK-E2F1-associated regulation; in cisplatin-resistant gastric cancer cells, JWA inhibits CK2-dependent XRCC1 phosphorylation and promotes XRCC1 ubiquitination and degradation, thereby reducing DNA-repair capacity and increasing cisplatin sensitivity. JWA also regulates Sp1 ubiquitination and degradation, p38 MAPK-associated cell-cycle arrest and apoptosis, and the AMPK-NEDD4L-EGFR pathway. In intestinal epithelial cells, JWA-associated ERK/FBXW7-NOTCH1/PPARγ/STAT5 signaling contributes to epithelial homeostasis and regeneration. JAC1 and JAC4 are shown as experimental JWA-inducing or JWA-activating compounds acting through YY1- and CTBP1-associated regulatory mechanisms, respectively. Ub, ubiquitin; P, phosphorylation; BER, base-excision repair; TNBC, triple-negative breast cancer. This figure was created using BioGDP (https://biogdp.com/zh).

## 3 Pathophysiological roles of JWA in disease and aging-related contexts

JWA has been implicated in multiple disease and tissue contexts, including malignant tumors, neurodegeneration, intestinal injury, and environmental stress responses. Across these settings, JWA-associated effects involve DNA damage repair, stress-responsive signaling, ferroptosis, autophagy, and tissue protection. However, the strength and direction of these relationships vary substantially according to cell type, disease state, stress intensity, and experimental model. In the context of aging, it is therefore important to distinguish direct evidence obtained from aging-specific models from findings derived from age-associated diseases and from mechanistic extrapolation. To avoid conflating disease-associated observations with direct evidence for biological aging, the evidence discussed below is considered at three levels: (i) direct aging evidence, including studies using aged tissues, aged animals, senescence models, or age-stratified human cohorts; (ii) evidence from diseases commonly associated with aging, including cancer, Parkinson’s disease, and intestinal dysfunction; and (iii) mechanistic extrapolation, referring to biologically plausible links between JWA-regulated stress responses and aging hallmarks that have not yet been directly demonstrated. This distinction is particularly important because aging-specific models, longitudinal datasets, and systematic tissue-level analyses of JWA remain limited.

### 3.1 JWA-associated roles in malignant tumors

JWA exhibits broad-spectrum tumor-suppressive properties in most malignancies, primarily by maintaining redox homeostasis, while exerting oncogenic effects in certain contexts. Its mechanisms involve regulating tumor cell proliferation, migration, differentiation, drug resistance, and immune responses via redox-sensitive pathways, with clinical significance in prognostic assessment, therapeutic efficacy prediction, and targeted therapy development (Ding et al., 2023a; Hu et al., 2025). Notably, JWA expression is significantly downregulated in elderly tumor patients, and its functional deficiency is closely associated with the occurrence, development and poor prognosis of age-related tumors (Wang et al., 2018; Li et al., 2022).

#### 3.1.1 Redox-associated regulation of tumor oncogenesis and progression

JWA regulates tumor progression through several context-dependent stress-response mechanisms involving MAPK signaling, cytoskeletal regulation, protein degradation, and DNA repair. JWA counteracts these processes via multiple redox-sensitive mechanisms. In metastasis inhibition, JWA regulates cancer cell migration through the MAPK-F-actin pathway and promotes CXCR4 degradation through the proteasome, thereby suppressing PI3K/AKT-associated invasion in breast cancer and hepatocellular carcinoma models. These mechanisms have been demonstrated in specific tumor contexts, whereas their direct dependence on redox status has not been established (Chen H. et al., 2007; Xu et al., 2018). In angiogenesis inhibition, JWA downregulates the NF-κB/STAT3/VEGF axis through the ING4-JWA-ILK pathway, thereby reducing angiogenic signaling and melanoma progression. These findings were obtained in tumor models associated with oxidative-stress signaling, but they do not establish with associated changes in redox-related signaling as the causal mediator of the ING4-JWA-ILK pathway (Lu et al., 2013a; Lu et al., 2013b). In proliferation and apoptosis regulation, JWA promotes tubulin polymerization through p38 MAPK signaling and enhances chemotherapy-induced apoptosis, whereas JWA deficiency is associated with altered redox homeostasis and increased tumorigenic phenotypes in specific cellular contexts (Chen et al., 2015; Qi and Li, 2018). JWA also regulates tumor differentiation: in acute promyelocytic leukemia, its expression synchronizes with ATRA-induced differentiation, enhancing induction therapy efficacy by modulating redox-sensitive differentiation pathways (Wei et al., 2001; Huang et al., 2006). Notably, JWA exhibits a dual “tumor suppressor-oncogene” pattern in leukemia and bladder cancer, related to tumor type, microenvironment, and redox context, and aging status is an important factor affecting this functional pattern (Hu et al., 2025; Hanahan, 2026).

#### 3.1.2 Redox-mediated tumor chemoresistance and immunity

JWA is a key regulator of this process: high JWA expression stabilizes XRCC1 to enhance BER pathway activity and DNA repair, inducing platinum resistance by enhancing XRCC1-associated DNA-repair capacity in tumor cells (Wang et al., 2012; Zhou et al., 2022). JWA-directed Pt (IV) prodrugs (e.g., Cis-wog, Cx-platin-Cl) reverse chemoresistance by inhibiting the JWA-XRCC1 pathway and reducing tumor-cell DNA-repair capacity. And regulating the tumor immune microenvironment (promoting effector T cell infiltration, inhibiting regulatory T cell recruitment) to achieve chemo-immunotherapy synergy (Gong et al., 2012; Chen et al., 2020). Additionally, JWA-related modulation of the CTBP1-JWA-AMPK/NEDD4L/EGFR axis has been reported to enhance the response of EGFR-mutant lung adenocarcinoma models to osimertinib. The contribution of redox changes to this sensitization remains unresolved, and the therapeutic relevance of this combination in older patients remains to be evaluated in age-specific preclinical and clinical studies (Wu X. et al., 2014; Ding et al., 2023b).

#### 3.1.3 Clinical associations and candidate biomarker evidence

Low JWA expression has been associated with metastasis, recurrence, and shorter overall survival in gastric cancer, hepatocellular carcinoma, esophageal cancer, and melanoma (Zhou et al., 2012; Wu X. et al., 2014). Co-expression of JWA with p53, murine double minute 2 (MDM2), XRCC1, or FAK has been associated with prognostic stratification in retrospective clinical analyses; in gastric cancer, the “low JWA + high FAK” combination has been associated with poorer prognosis and differential treatment outcomes in patients receiving fluorouracil-leucovorin-oxaliplatin chemotherapy (Chen et al., 2013). These findings support the potential value of JWA-related expression patterns for retrospective prognostic and treatment-response analyses, but their incremental clinical utility requires prospective validation.

JWA expression has also been investigated in relation to sensitivity or response to platinum-based chemotherapy, ATRA differentiation therapy, and radiation therapy in selected experimental or clinical contexts. These observations may reflect differences in tumor-cell stress-response and DNA-repair states, but they do not establish JWA expression as a validated predictor of treatment sensitivity or a treatment-selection tool. Similarly, JWA polymorphisms, including −76G>C and 723T>G, have been investigated as candidate susceptibility markers for bladder cancer and leukemia; their potential value for risk assessment requires prospective validation JWA-activating compounds such as JAC1 and JAC4, as well as JWA-targeted agents including Cis-wog and recombinant JWA, have been investigated in preclinical tumor models. These approaches provide experimental evidence for JWA modulation as a candidate intervention strategy, but their therapeutic efficacy, safety, and disease- or age-specific applicability remain to be established (Zhai et al., 2022; Ding et al., 2023b).

### 3.2 JWA-associated mechanisms in neurodegenerative disease-relevant models

JWA has been investigated in several neurodegenerative disease models, particularly Parkinson’s disease and prion disease, where oxidative stress, ferroptosis, protein aggregation, and proteostasis are prominent pathological features JWA plays a critical regulatory role in these diseases via maintaining glutamate homeostasis, inhibiting abnormal protein aggregation, regulating autophagy, and resisting oxidative stress, mechanisms associated with oxidative-stress responses, thus providing an important molecular target for intervention in age-related neurodegenerative diseases (Siddique et al., 2023; Zhao et al., 2024).

#### 3.2.1 Redox-mediated protection in Parkinson’s disease

JWA expression is synchronously downregulated with dopaminergic neuron marker tyrosine hydroxylase (TH) in Parkinson’s disease patient brain tissues and model mice, indicating impaired redox regulation; JWA deficiency exacerbates ferritinophagy and ferroptosis, accelerating dopaminergic neuron degeneration, while JWA overexpression or JWA-activating compound JAC4 activation inhibits ferritinophagy via NCOA4 binding, reducing Fe^2+^ accumulation and lipid peroxidation, improving motor symptoms and brain dopamine levels in Parkinson’s disease model mice (Zhao et al., 2024; Sawamoto et al., 2025; Wu et al., 2025). JWA eliminates α-synuclein aggregates by inducing autophagy: it directly binds autophagy-related gene 12 (ATG12), inhibits its ubiquitination and degradation, promotes formation of the autophagy-related gene 5 (ATG5)-ATG12 complex, and enhances autophagic flux to alleviate α-synuclein-mediated protein toxicity, an important redox-sensitive process critical for clearing age-related α-synuclein aggregates (Hallacli et al., 2022; Siddique et al., 2023; Ahanger and Dar, 2024). In astrocytes, JWA upregulates GLT-1 expression via the ERK/AKT/CREB pathway, enhancing glutamate uptake and mitigating excitotoxicity; its deficiency exacerbates 1-methyl-4-phenyl-1,2,3,6-tetrahydropyridine (MPTP)/paraquat-induced neurodegeneration by disrupting redox balance (Wang et al., 2018). Additionally, JWA antagonizes paraquat-induced oxidative stress via activating the MEK/ERK/PI3K/AKT/Nrf2 pathway, delaying neuron loss and motor dysfunction (Zhao et al., 2017). JWA expression has been reported to be reduced in dopaminergic neurons in Parkinson’s disease-relevant human tissues and experimental models, and experimental manipulation of JWA influences ferritinophagy, ferroptosis, and neuronal injury in Parkinson’s disease models. These findings provide disease-associated evidence for a role of JWA in Parkinson’s disease pathology. However, the available evidence does not establish that JWA dysregulation is specifically caused by biological aging or that the observed effects constitute a general mechanism of neuronal aging. Therefore, the relevance of the JWA-NCOA4-ferritinophagy axis to age-dependent neurodegeneration should be considered a disease-associated mechanism with potential aging relevance rather than direct evidence of an aging mechanism (Ryan et al., 2013; Yao et al., 2024; Zhao et al., 2024).

#### 3.2.2 JWA-associated regulation of glutamate homeostasis and excitotoxicity

JWA participates in neuroprotection via regulating glutamate transporters: it specifically binds EAAC1, modulating EAAC1-mediated glutamate transport and cysteine uptake to maintain GSH levels and redox balance, and its downregulation leads to glutamate homeostasis disruption and disrupted GSH homeostasis. (Lin et al., 2001; Butchbach et al., 2002; Sedlak et al., 2019). Abnormal JWA expression disrupts glutamate homeostasis and GSH synthesis, enhancing neuronal sensitivity to oxidative stress and contributing to epilepsy and Alzheimer’s disease pathogenesis (Butchbach et al., 2003). Targeted JWA regulation or blocking its excitatory amino acid transporter 3 (EAAT3) binding restores glutamate transport balance and alleviates excitotoxicity-associated damage while restoring glutathione and redox homeostasis (Butchbach et al., 2002). Additionally, JWA selectively regulates glutamate and taurine homeostasis in PC12 cells, avoiding glutamate-induced excitotoxicity while preserving taurine’s neuroprotective effect; both processes are redox-sensitive (Li et al., 2003).

#### 3.2.3 Role in other redox-dysregulated neurodegenerative diseases

Prion protein (PrP) misfolding (PrPˢ^c^) triggers fatal neurodegenerative diseases, which are also associated with aging, characterized by abnormal protein aggregation and redox dysregulation (An et al., 2025; Peng et al., 2025). Misfolded PrP induces normal PrP conformational changes, forming toxic aggregates; the disease is highly infectious with 100% mortality and no effective therapies (Neumann et al., 2024; An et al., 2025; Long et al., 2025). In prion disease, JWA induces reticulophagy/ER-phagy via the Ca^2+^-AMPK pathway, specifically clearing ER-aggregated misfolded PrPˢ^c^, alleviating ER stress and neuronal damage by reducing redox stress. JWA overexpression reverses PrPˢ^c^-mediated cytotoxicity, and this autophagy-regulated abnormal protein clearance mechanism also applies to α-synuclein, suggesting that related JWA-associated proteostasis mechanisms may warrant investigation in other protein-aggregation disorders sharing related stress-response mechanisms (Benedetti et al., 2025; Kamble et al., 2025; Liu Z. et al., 2025; Veselic et al., 2025). Cognitive function relies on hippocampal neurogenesis and synaptic plasticity, which are redox-sensitive; abnormalities induce cognitive decline (typical in Alzheimer’s disease, the most common age-related neurodegenerative disease) (Liu Z. et al., 2025; Veselic et al., 2025). JWA deficiency enhances hippocampal neurogenesis and synaptic plasticity via the FAK/PI3K/AKT/mTOR pathway, improving spatial cognitive function by normalizing redox balance. It is speculated that JWA overexpression may inhibit neurogenesis and exacerbate cognitive impairment, this finding raises a hypothesis that JWA-dependent regulation of hippocampal neurogenesis may differ from its protective effects observed in Parkinson’s disease-relevant models; however, whether JWA directly contributes to Alzheimer’s disease pathogenesis or represents a therapeutic target remains unknown (Sha et al., 2016). These findings indicate that JWA does not exert a uniformly beneficial or detrimental effect on neuronal function. Instead, its functional direction may depend on cell type, brain region, disease context, developmental or aging state, basal stress level, and pathway dependence. In Parkinson’s disease-relevant models, increased JWA activity is associated with protection against oxidative stress, ferritinophagy, and ferroptosis, whereas reduced JWA activity in the hippocampus can enhance neurogenesis and synaptic plasticity through FAK/PI3K/AKT/mTOR signaling. The biological switch underlying these context-specific effects remains unresolved. The distinct neuronal contexts and JWA-associated effector programs described above are summarized in Figure 3.

**FIGURE 3:**
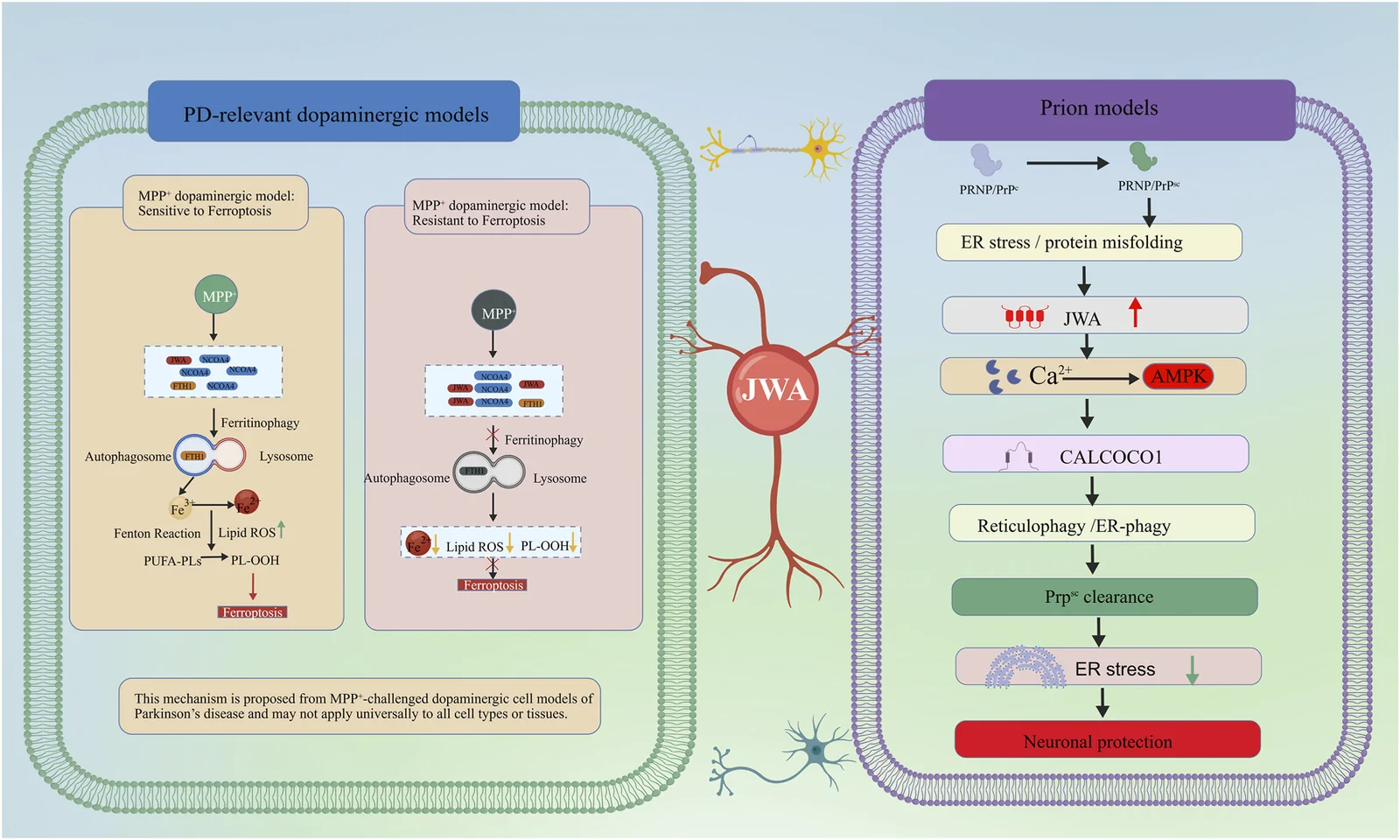
Context-specific neurodegenerative mechanisms of JWA. JWA-associated neuroprotective mechanisms are presented in two experimentally distinct neuronal contexts. In Parkinson’s disease-relevant dopaminergic models, JWA interacts with NCOA4 and suppresses NCOA4-FTH1-dependent ferritinophagy, thereby limiting iron accumulation, lipid peroxidation, and ferroptotic neuronal injury. JWA induction by JAC4 or genetic overexpression enhances this protective pathway. In prion models, JWA-associated Ca^2+^-AMPK signaling and interaction with the reticulophagy receptor CALCOCO1 promote ER-associated protein clearance and alleviate proteostatic stress. These pathways represent context-specific mechanisms rather than a single universal neuroprotective program. This figure was created using BioGDP (https://biogdp.com/zh).

### 3.3 JWA-associated tissue homeostasis and stress responses

#### 3.3.1 Intestinal homeostasis, aging, and radiation protection

JWA regulates intestinal stem-cell and epithelial homeostasis through the ERK/FBXW7/NOTCH1/PPARγ/STAT5 axis. Available evidence supports a role in oxidative-stress-associated intestinal injury, whereas its direct contribution to biological intestinal aging remains incompletely defined (Li et al., 2022). Radiation-induced intestinal injury impairs mucosal epithelium integrity, triggers inflammation and hemorrhage, and inhibits intestinal stem cell regeneration, all associated with excessive oxidative stress (Jena et al., 2024; Myint et al., 2025). The JWA-activating compound JAC4 protects against radiation-induced intestinal injury via JWA-associated antioxidant and anti-inflammatory signaling, providing preclinical evidence for further investigation of JWA activation in normal-tissue protection during radiotherapy (Zhu et al., 2006; Zhou et al., 2022).

#### 3.3.2 Cellular response to redox-active environmental pollutants

In response to Cd and B [a]P exposure, JWA participates in ROS-MAPK-associated stress responses and cellular damage control (Wang Z. et al., 2020; Saleem et al., 2025). As an environmental stress-responsive regulator, JWA responds to Cd and B [a]P exposure and participates in the regulation of cellular damage and apoptosis through the ROS-MAPK pathway (Cao et al., 2007; Chen R. et al., 2007). Its expression level reflects the degree of pollutant-induced cellular redox damage, providing a molecular basis for environment-related disease risk assessment. Importantly, persistent exposure to redox-active environmental stressors can promote cellular dysfunction, raising the possibility that JWA-mediated stress responses may intersect with aging-related processes. However, a direct role for JWA in environmentally accelerated aging has not been established.

#### 3.3.3 JWA-associated mechanisms in other redox-related diseases

JWA has also been investigated in cardiovascular injury, bone metabolism, and other redox-related conditions. Because several of these disorders become more prevalent with advancing age, their JWA-associated mechanisms may be relevant to aging-related disease biology; however, age-specific JWA mechanisms remain insufficiently characterized (Ikemoto et al., 2002; Wu et al., 2015; Hu, 2025; Kirk et al., 2025; Yan et al., 2025). For example, JWA-associated regulation of vascular endothelial repair has been linked to oxidative-stress responses in OSAHS-related cardiovascular complications, but the causal contribution of redox changes and the therapeutic relevance of JWA modulation remain to be established (Liu et al., 2022; Yan et al., 2025). Taken together, the disease-specific evidence supports a context-dependent model of JWA rather than a uniform disease-modifying function. Across cancer, neurodegenerative disease, and intestinal injury, the most consistently supported JWA-associated mechanisms involve DNA repair, stress-responsive MAPK signaling, iron/ferroptosis regulation, glutathione homeostasis, and autophagic or ER-phagic processes. However, the strength of evidence differs substantially across disease contexts, and the extension of these mechanisms to biological aging remains partly inferential. This distinction provides the basis for the evidence-weighted aging framework presented (Figure 4) and the evidence summary and development potential of major JWA-associated mechanistic modules (Table 1).

**FIGURE 4:**
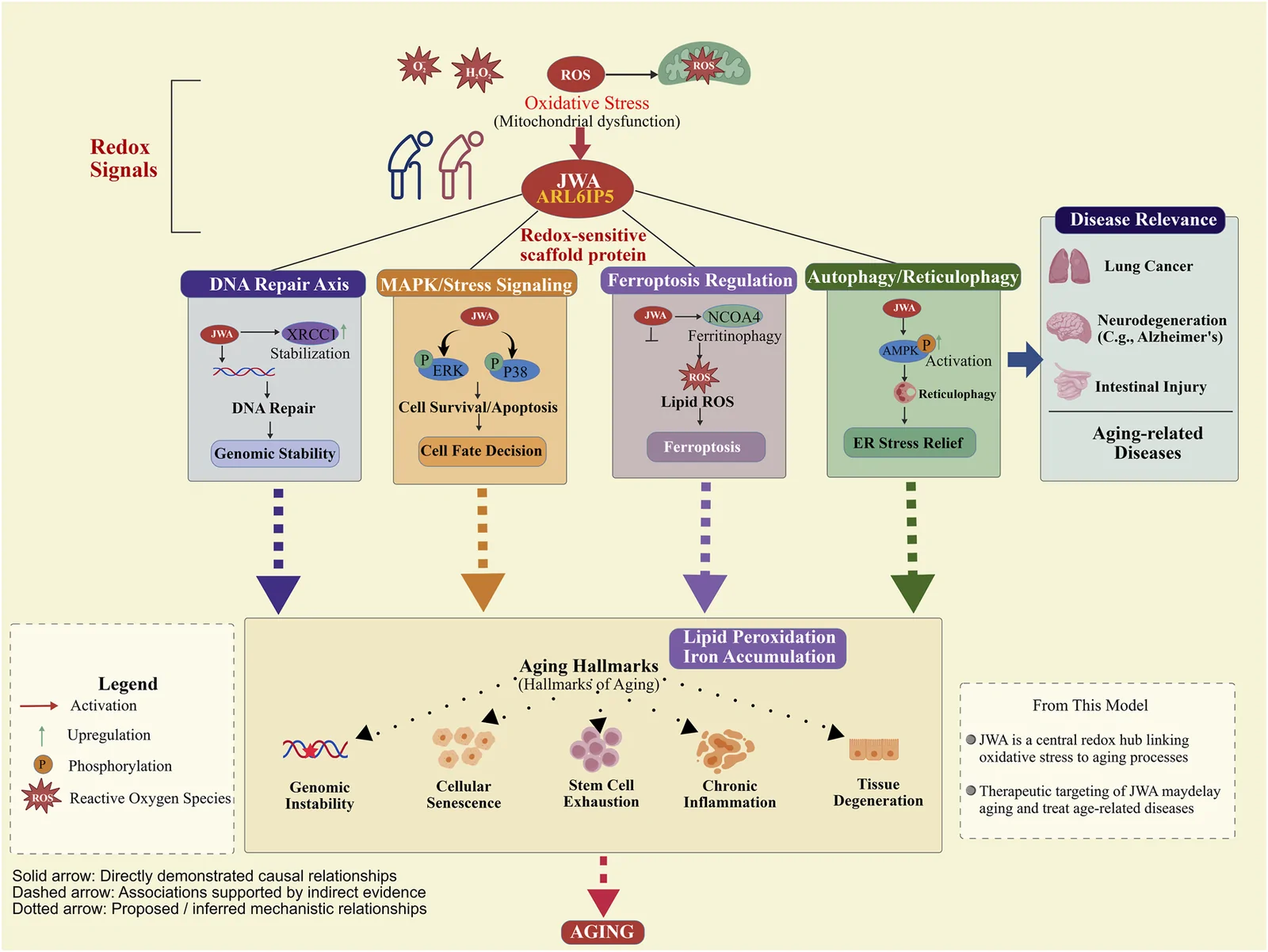
Evidence-weighted conceptual framework linking JWA-mediated redox regulation to aging hallmarks and age-associated diseases. JWA-associated effector modules, including DNA repair, MAPK stress signaling, ferroptosis regulation, and autophagy/reticulophagy, are integrated with aging hallmarks and disease phenotypes according to the current strength of evidence. Solid arrows indicate relationships supported by direct experimental evidence; dashed arrows indicate indirect or context-dependent evidence; dotted arrows indicate proposed or inferred relationships; and question marks indicate unresolved mechanisms. The framework represents a conceptual integration of current JWA evidence rather than a uniformly established causal network. This figure was created using BioGDP (https://biogdp.com/zh).

**TABLE 1:** Evidence summary and development potential of major JWA-associated mechanistic modules.

Mechanistic module	Representative mechanisms	Research status	Major limitations	Development potential
DNA damage repair	JWA-XRCC1/BER; context-dependent CK2-XRCC1 regulation	Direct mechanistic evidence in oxidative-stress and cancer models	Context-dependent regulation; limited aging-specific validation	Mechanistic basis for investigating DNA-damage responses and therapy resistance
Redox and stress adaptation	MAPK signaling; EAAC1/GSH; NCOA4/ferritinophagy and ferroptosis	Mechanistically supported in selected cellular and animal models	Mechanisms vary across tissues and disease contexts	Candidate framework for further studies of stress adaptation and ferroptosis-related intervention
Proteostasis and tissue homeostasis	ER-phagy/reticulophagy; ERK/fbxw7-notch1 signaling; intestinal epithelial protection	Emerging experimental evidence	Limited direct aging-specific evidence and translational validation	Hypothesis-generating framework for tissue protection and age-associated disease research

## 4 Translational implications of JWA-mediated redox regulation in aging and age-related diseases

### 4.1 JWA as a potential biomarker of aging-related redox dysfunction

JWA expression, gene polymorphisms, and combinations with downstream proteins have been investigated as candidate biomarkers for disease classification, prognosis, and treatment-response assessment. However, most available studies remain exploratory or retrospective, and prospective multicenter validation, particularly in aging populations, is limited (Butchbach et al., 2002; Zhang et al., 2026).

#### 4.1.1 JWA-related biomarker assays in tumors

Key detection approaches used in JWA-related biomarker studies include PCR-RFLP, RT-qPCR, western blotting, and immunohistochemistry (IHC). PCR-RFLP has been used to characterize JWA polymorphisms (−76G>C, 454C>A, and 723T>G) and their associations with disease susceptibility; whether these variants can serve as susceptibility markers for age-related tumors requires prospective validation (Li et al., 2007; Shen et al., 2007). RT-qPCR has been used to quantify JWA expression in experimental and disease-associated studies, whereas the value of JWA expression for early detection or risk assessment of age-related tumors remains to be prospectively validated (Qi and Li, 2018). Western blotting has similarly been used to assess JWA protein abundance and associated pathway changes in research settings, but its clinical utility for treatment selection or response monitoring has not been established. IHC has been used to evaluate tissue JWA expression and its associations with clinicopathological and prognostic features; however, standardized assays and prospective validation are still required before clinical implementation (Zhai et al., 2022; Ding et al., 2023b).

Regarding clinical associations, JWA-related biomarker findings can be considered at several levels. For risk assessment, JWA expression measured by RT-qPCR or western blotting has been investigated in relation to malignant transformation and disease-associated redox imbalance, but its value for predicting cancer risk, particularly in aging tissues, remains to be established prospectively (Qi and Li, 2018). For prognosis, IHC-based JWA expression has been associated with clinicopathological features and survival outcomes in esophageal squamous cell carcinoma, hepatocellular carcinoma, and melanoma, with lower JWA expression reported in association with tumor progression and poorer outcomes (Zhou et al., 2012; Wu X. et al., 2014); Combined detection of JWA with p53, MDM2, XRCC1, or FAK has also been associated with prognostic stratification in gastric cancer; however, whether such combinations provide incremental clinical utility, particularly in older patients, requires prospective validation (Chen et al., 2013).

JWA-related measurements have also been investigated in the context of treatment response. RT-qPCR and western blot analyses of JWA and XRCC1 expression have been associated with platinum-based chemotherapy response or resistance in selected experimental and clinical contexts, potentially reflecting differences in oxidative-stress-associated DNA-repair capacity (Chen et al., 2020; Wang X. et al., 2020). Combined assessment of JWA and breast cancer susceptibility gene 1 (BRCA1) mRNA has likewise been investigated in relation to cisplatin or docetaxel response in advanced esophageal squamous cell carcinoma (Wei et al., 2015). These findings support the potential value of JWA-related measurements as candidate biomarkers of disease characteristics or treatment response, but they do not yet establish their clinical utility for screening, treatment selection, or response-guided management, including in older patients.

#### 4.1.2 JWA-related measurements in neurodegenerative disease models

The reported JWA/TH expression pattern in Parkinson’s disease provides disease-associated evidence, but age-stratified validation is required to determine whether this association is specifically modified by patient age (Zhao et al., 2024). JWA and GLT-1 expression analyses have been investigated in relation to dopaminergic neuronal injury, glutamate homeostasis, and oxidative-stress responses in experimental models (Wang et al., 2018). These findings support the use of JWA-related measurements as research readouts of neuronal stress and injury, but their value as diagnostic biomarkers of age-related dopaminergic neurodegeneration has not been established. For prion disease, RT-qPCR and western blot analyses of JWA and downstream molecules, including CALCOCO1, PrPˢ^c^, and ATG12, have been used to characterize abnormal protein aggregation, ER-phagy/reticulophagy, and associated stress responses (Kamble et al., 2025). These measurements currently support mechanistic investigation rather than clinical diagnosis, and their diagnostic value in other protein-aggregation diseases requires independent clinical validation.

#### 4.1.3 JWA-related measurements in other redox-related diseases

RT-qPCR or western blot detection of JWA in intestinal tissue or peripheral blood has been investigated in relation to radiotherapy-associated intestinal injury and responses to protective interventions, potentially reflecting changes in cellular stress responses (Zhou et al., 2022). These measurements may provide research readouts of intestinal injury and stress responses, but their value for predicting susceptibility, evaluating disease severity, or monitoring treatment efficacy has not been clinically established. Analysis of JWA and ROS/MAPK pathway molecules has been used to characterize cellular responses to Cd-induced cytotoxicity and environmental pollutant exposure (Cao et al., 2007). Similarly, detection of JWA and MEK/ERK/Elk pathway components has been investigated in relation to cellular responses to chemical carcinogens and chemically induced skin cancer risk (Gong et al., 2012). These observations provide experimental or disease-associated evidence, whereas their value for individual risk prediction or clinical decision-making remains to be determined. JWA has also been associated with pathological processes in osteoporosis and cardiovascular disease through effects on bone metabolism and vascular endothelial homeostasis, but age-specific biomarker utility remains insufficiently characterized (Wu et al., 2015; Yan et al., 2025).

### 4.2 Therapeutic strategies targeting JWA-associated stress and signaling pathways

Several experimental strategies have been investigated to modulate JWA-associated stress and signaling pathways, including JWA-inducing compounds, pathway-directed interventions, combination approaches, and targeted delivery systems (Wang X. et al., 2020; Zhai et al., 2022; Ding et al., 2023b). However, the available evidence remains predominantly preclinical and varies substantially across disease models. To maintain an evidence-calibrated interpretation, the following discussion distinguishes three levels of translational evidence: experimentally demonstrated interventions, preclinical candidate strategies, and mechanistic therapeutic hypotheses. Experimentally demonstrated interventions refer to effects directly supported by intervention and outcome measurements in disease-relevant models; preclinical candidate strategies are approaches supported by disease-model evidence but requiring further validation of efficacy, safety, mechanism, or generalizability; and mechanistic therapeutic hypotheses are proposed applications inferred from JWA-associated mechanisms that have not yet been directly tested in the relevant disease context.

#### 4.2.1 Tumor-targeted therapeutic strategies

For chemotherapy sensitization in low-JWA-expressing tumors, JAC1, JAC4, or modulation of MEK/ERK/p38-associated signaling has shown enhanced apoptotic responses when combined with chemotherapeutic agents in experimental models (Chen H. et al., 2007; Zhou et al., 2008; Zhai et al., 2022). These findings support candidate preclinical strategies for further evaluation in age-relevant tumor models rather than established treatment approaches. In platinum-resistant tumors with high JWA expression, JWA-targeted Pt(IV) prodrugs, including Cis-wog and Cx-platin-Cl, have been investigated for reversing chemoresistance through modulation of the JWA-XRCC1 pathway and associated tumor-cell responses (Chen et al., 2020; Wang X. et al., 2020). Their efficacy, safety, pharmacokinetics, and disease-specific applicability require further preclinical validation. In metastasis-related models, JWA-associated interventions targeting MAPK/F-actin, CXCR4, or FAK-related signaling have shown inhibitory effects on invasion or migration in experimental systems (Chen H. et al., 2007; Wu X. et al., 2014; Xu et al., 2018). These observations provide a mechanistic basis for future preclinical investigation but do not currently support JWA-guided treatment selection or clinical risk stratification (Chen et al., 2013).

#### 4.2.2 Therapeutic strategies for neurodegenerative diseases

For Parkinson’s disease-relevant models, JWA activation, including JAC4 treatment, has been associated with protection of dopaminergic neurons through suppression of ferritinophagy, modulation of Nrf2-associated responses, or enhancement of GLT-1-mediated glutamate uptake (Zhao et al., 2017; Wang et al., 2018; Zhao et al., 2024). Combination approaches involving ferroptosis inhibitors or autophagy modulators have also shown protective effects in experimental models. These findings support candidate preclinical strategies for further investigation rather than an established therapeutic strategy for age-related Parkinson’s disease (Siddique et al., 2023; Zhao et al., 2024). For prion disease, JWA activation or modulation of the Ca^2+^-AMPK-CALCOCO1 pathway has been associated with enhanced reticulophagy and PrPˢ^c^ clearance in disease models (Kamble et al., 2025). This mechanism may warrant investigation in other protein-aggregation disorders sharing related stress-response mechanisms, but its broader therapeutic applicability remains hypothetical. In hippocampal neurogenesis models, JWA downregulation or inhibition has been reported to activate the FAK/PI3K/AKT/mTOR pathway and promote neurogenesis and synaptic plasticity. This represents a context-dependent neuronal phenotype; Alzheimer’s disease-specific studies involving direct JWA manipulation remain lacking, and no Alzheimer’s disease-specific therapeutic implication can currently be established (Sha et al., 2016).

#### 4.2.3 Therapeutic strategies for other redox-related diseases

In radiation injury models, JAC4 treatment has been associated with protection against radiation-induced intestinal injury through JWA-associated antioxidant and anti-inflammatory responses (Zhou et al., 2022). These findings support further preclinical evaluation of JWA activation for normal-tissue protection during radiotherapy, including assessment in age-relevant models. In environmental-stress models, JWA-associated interventions have been reported to enhance cellular responses to heavy metals such as Cd and chemical carcinogens such as B[a]P, whereas the therapeutic efficacy of these approaches in age-related or systemic disease models remains to be established (Cao et al., 2007; Chen R. et al., 2007). In intestinal disease models, JWA activation or modulation of the ERK/FBXW7-NOTCH1 axis has been associated with improved epithelial repair and homeostasis (Li et al., 2022). Whether these effects translate into a strategy for preventing or delaying age-related intestinal dysfunction remains a mechanistic therapeutic hypothesis requiring validation in aging-specific models.

#### 4.2.4 Drug optimization and precision delivery for redox targeting

At the drug-development level, structure-guided optimization of JWA-modulating compounds such as JAC1 and JAC4 should be considered only after their direct compound-target relationships and binding determinants have been experimentally resolved. Current evidence supports modulation of YY1/CTBP1-JWA-associated pathways, but the structural basis required for definitive structure-guided optimization remains incompletely defined (Zhai et al., 2022; Ding et al., 2023b).; PRA1-domain-targeted compounds may represent a future drug-development strategy because of the potential role of the PRA1 domain in ER membrane organization and ER-phagy; however, their direct target engagement, pharmacological activity, and therapeutic efficacy remain to be established (Yamamoto and Sakisaka, 2025b; Yamamoto and Sakisaka, 2025a). Tumor-targeted nanocarriers, brain-targeted systems, and intestinal-targeted delivery approaches may likewise be explored as preclinical strategies for JWA modulation, but their biodistribution, target engagement, efficacy, and safety require experimental validation before translational development can be considered (Alonso-Valenteen et al., 2025; Jin et al., 2025). Importantly, the therapeutic direction of JWA modulation should not be assumed to be universally activating or inhibitory. The evidence reviewed here indicates that JWA may preserve XRCC1-dependent DNA repair in one cellular context but facilitate drug resensitization through XRCC1 destabilization in another, while JWA activation is associated with neuroprotection in some disease-relevant models, whereas JWA loss enhances hippocampal neurogenesis in a different experimental context. Thus, the therapeutic question is not simply whether JWA should be increased or decreased. Rather, future intervention should be guided by cell state, disease context, basal stress/redox state, DNA-repair demand, proteostatic burden, and the specific downstream effector program being targeted. This context-dependent principle should be established before JWA modulation is considered for disease-specific therapeutic development.

## 5 Research challenges and future perspectives

### 5.1 Core challenges in JWA redox regulation research

#### 5.1.1 Incomplete elucidation of JWA-associated redox mechanisms

Key JWA-associated redox mechanisms remain unresolved: direct binding sites, atomic structures, and regulatory patterns between JWA and redox-sensitive targets (MAPK molecules, NCOA4, XRCC1) are undefined, these structural uncertainties also limit the extent to which current JWA-modulating compounds can be subjected to definitive structure-guided optimization (Li et al., 2003; Qiu et al., 2018); the tubulin-binding domain and microtubule polymerization regulatory mechanisms in redox signaling are uncharacterized (Li et al., 2003). Functionally, key regulatory factors governing JWA’s bidirectional stress-response roles (tumor-suppressive vs. oncogenic) are unknown (Hu et al., 2025); the molecular mechanisms underlying cell-type-specific MAPK subtype regulation in redox signaling remain unclear (Wu Y.-Y. et al., 2014; Chen et al., 2015). Additionally, how JWA polymorphisms influence redox stress sensitivity (e.g., effects on protein structure and promoter redox responsiveness) and the balanced regulatory network of JWA ubiquitination in redox signaling (e.g., deubiquitinating enzyme identity at the K158 site) have not been fully addressed (Li et al., 2007; Qiu et al., 2018). More importantly, the molecular mechanisms by which JWA regulates aging hallmarks and how aging affects JWA’s redox regulatory function are still unclear, which is a key gap in current research (Yan et al., 2025). The age-dependent post-translational modification of JWA and its impact on protein stability and downstream pathway regulation have not been systematically explored, and the crosstalk between JWA and other classic aging-related signaling pathways (e.g., mTOR, SIRT1) remains to be elucidated, which restricts the understanding of JWA’s core role in the aging regulatory network (Iyer et al., 2024; Saeedi Saravi et al., 2025; Yan et al., 2025).

#### 5.1.2 Deficient clinical translation of redox-targeted approaches

JWA-related clinical translation remains limited due to insufficient clinical validation and incomplete characterization of its context-dependent molecular functions, and the aging-related clinical research is even more scarce, which severely limits its application in age-related disease intervention. In biomarker research: Most studies are single-center, small-sample, and retrospective, lacking multi-center, multi-ethnic prospective validation of JWA and combined redox-sensitive biomarkers (p53, MDM2, XRCC1) (Liu et al., 2012; Wang et al., 2012; Chen et al., 2013; Wei et al., 2015); clinical data on JWA expression correlation with redox dysregulation in disease progression/chemotherapeutic efficacy (e.g., Parkinson’s disease) remain scarce, and there is almost no large-sample clinical data on JWA as a biomarker for age-related disease risk stratification and prognostic evaluation, although age-stratified clinical validation remains limited (Chen et al., 2005; Wang et al., 2018; Siddique et al., 2023; Zhao et al., 2024). In therapeutic translation: Human pharmacokinetics, long-term safety, and optimal dosages of JWA-targeted redox modulators (JAC1, JAC4, Cis-wog) are undetermined, with no candidates entering clinical development (Wang X. et al., 2020; Zhai et al., 2022; Ding et al., 2023b); the therapeutic effect and safety of JWA-activating compound in the elderly population have not been verified by preclinical or clinical studies, and the age-related pharmacokinetic changes of JWA-targeted drugs are still unclear; JWA-related gene therapy and targeted delivery technologies are in preclinical stages, lacking *in vivo* efficacy and safety verification (Chen H. et al., 2007; Maier et al., 2009); JWA’s causal role in redox dysregulation-related diseases remains unclear, hindering its direct translation as a clinical intervention target (Butchbach et al., 2002). In addition, the combination strategy of JWA-targeted therapy with traditional anti-aging interventions (e.g., caloric restriction, antioxidant supplementation) has not been explored, and there is a lack of research on personalized JWA-targeted intervention based on age and genetic background.

#### 5.1.3 Lack of standardization in redox-related research models and detection technologies

The current research on JWA and aging is further limited by the lack of aging-specific standardized models and detection methods. In research models: Most mechanism studies rely on single cell lines (e.g., HeLa, PC12, K562), lacking validation in primary cells, humanized animal models, and disease subtypes under different redox contexts (Chen et al., 2005; Chen H. et al., 2007; Zhou et al., 2022; Siddique et al., 2023); key JWA redox functions (e.g., radiation protection, neuroprotection) are only validated in mouse models, with no systematic verification in aged animal models and human aging tissue samples (Wang et al., 2018; Zhou et al., 2022); there is a lack of conditional knockout animal models of JWA in specific tissues of aged mice, which cannot accurately simulate the tissue-specific regulatory role of JWA in the aging process. In detection technologies: JWA expression detection methods (IHC, qPCR) lack unified standards for antibody selection, staining scoring, and primer design, leading to poor inter-study result comparability in redox-related studies; there is no standardized detection framework for JWA expression across age-stratified human blood, tissue, and aging-cell samples, which restricts the clinical application of JWA as an aging-related biomarker; gene polymorphism studies are severely limited to the southern Chinese Han population, lacking large-sample validation across ethnicities/regions and different age groups (Li et al., 2007; Shen et al., 2007). Additionally, insufficient focus on JWA interactions with key redox pathways (e.g., mTOR, gut microbiota-related redox pathways) in the aging microenvironment limits understanding of its regulatory network in complex physiological/pathological microenvironments (Li et al., 2022; Zhou et al., 2022).

#### 5.1.4 Insufficient breadth and depth of redox-related functional research

In terms of breadth: Regulatory networks of JWA in emerging redox-related fields (ER autophagy, iron metabolism, amino acid homeostasis) are incompletely elucidated; for example, molecular kinetic mechanisms of JWA-mediated ER membrane remodeling and specific stress-responsive transport pathways regulating taurine/glutamate homeostasis remain unclear (Li et al., 2003; Yamamoto and Sakisaka, 2025b; Zhang et al., 2026); functional studies in non-neural/non-tumor redox-related fields (e.g., bone metabolism, drug addiction) are fragmented (Ikemoto et al., 2002; Wu et al., 2015), and the research on JWA’s role in age-related cardiovascular diseases, and immune senescence is still in the preliminary stage, with no systematic exploration of its core regulatory mechanisms. In terms of depth: Downstream redox-sensitive target genes and key signaling nodes of JWA have not been fully identified; core mechanisms, including the E3 ubiquitin ligase mediating Sp1 degradation and detailed Nrf2 pathway activation processes in redox signaling, remain undefined (Chen et al., 2014; Zhao et al., 2017); research on JWA’s long-term regulatory effects and molecular mechanisms in aging-related redox dysregulation and environmental pollutant responses is scarce (Li et al., 2022; Yan et al., 2025), and the dynamic changes of JWA expression and function during the entire aging process (from young to old) and its correlation with aging-related physiological and pathological changes have not been clarified. In addition, the sex-specific and tissue-specific differences in JWA’s redox regulatory function during aging have not been explored, and the impact of environmental factors (e.g., diet, exercise, environmental pollutants) on JWA expression in the aging process and its underlying mechanisms are still unclear.

### 5.2 Future research directions focused on redox regulation

#### 5.2.1 In-depth elucidation of JWA-associated redox mechanisms linked to aging

For JWA research, the future focus should be on integrating aging research into the core of molecular mechanism elucidation, and systematically exploring the crosstalk between JWA and aging-related signaling pathways. For protein interactions and structural bases, crystal structure analysis and cryo-electron microscopy should be employed to clarify JWA’s core binding sites, atomic structures, and regulatory patterns with redox-sensitive target proteins (NCOA4, XRCC1, MAPK pathway molecules) (Wang et al., 2009; Shen et al., 2011; Zhao et al., 2024); resolve the amino acid residues mediating JWA-NCOA4 binding and the molecular network regulating ferritinophagy inhibition, and explore the age-dependent changes of this binding interaction (Li et al., 2003; Zhao et al., 2024); define the key JWA-tubulin binding domain and detailed regulatory mechanisms of microtubule polymerization in redox signaling (Li et al., 2003); elucidate the balanced regulatory mechanisms of JWA post-translational modifications (ubiquitination, phosphorylation) in redox responses, and clarify the age-dependent changes of JWA post-translational modification and its impact on protein stability (Qiu et al., 2018). For functional regulatory networks and specificity mechanisms, systematically decode the switching mechanism of JWA’s bidirectional stress-responsive functions, identifying key regulatory factors (e.g., tumor microenvironment, redox context, aging status) and downstream signaling nodes (Hu et al., 2025); explore the molecular mechanisms underlying JWA’s cell-type-specific and age-dependent regulation of MAPK subtypes in redox signaling (Wu Y.-Y. et al., 2014; Chen et al., 2015); decipher the functional mechanisms by which JWA gene polymorphisms (−76G>C, 723T>G) influence redox stress sensitivity and disease risk in different age groups (Li et al., 2007; Shen et al., 2007); focus on elucidating the molecular mechanisms of JWA regulating aging hallmarks such as genomic instability, cellular senescence, and stem-cell dysfunction, and explore the crosstalk between JWA and classic aging-related signaling pathways (mTOR, SIRT1, AMPK, etc.) in the redox regulatory network. In addition, explore the age-dependent epigenetic regulation mechanism of JWA (DNA methylation, histone modification) and its impact on gene expression and redox regulatory function.

#### 5.2.2 Clinical translation and application expansion of redox-targeted approaches for age-related diseases

For JWA research, the future clinical translation should focus on the elderly population and age-related diseases, and prioritize validation of JWA as a candidate biomarker and experimental therapeutic target through appropriately designed preclinical and clinical studies.

For biomarker clinical validation and standardization: Conduct multi-center, multi-ethnic, large-sample prospective clinical studies to evaluate the reproducibility and incremental value of JWA-associated biomarkers and its combined redox-sensitive biomarkers (p53, MDM2, XRCC1) in prognostic prediction, efficacy evaluation and age-stratified risk assessment for age-associated diseases (Liu et al., 2012; Wang et al., 2012; Ye et al., 2013); across age-stratified human peripheral blood, tissue samples, and relevant aging-cell models, unifying antibody selection, staining scoring criteria, and primer design to improve the comparability and reliability of results (Chen et al., 2013; Ye et al., 2013); expand clinical validation of JWA gene polymorphisms, in different age groups and ethnicities, exploring their synergistic effects with environmental redox factors (e.g., smoking, chemical toxin exposure, diet, exercise) (Li et al., 2007; Shen et al., 2007). Future studies may evaluate whether JWA expression and genotype add predictive value to established clinical risk models based on JWA expression level and gene polymorphism, and verify its clinical application value in the early screening and prognostic evaluation of age-related diseases (tumor, neurodegenerative diseases, cardiovascular diseases).

For targeted drug development and translation: Further characterize existing JWA-modulating compounds (JAC1, JAC4, and Cis-wog) with respect to direct target relationships, pharmacological selectivity, pharmacokinetics/pharmacodynamics, toxicity, dose-response relationships, and disease-specific efficacy. These studies should proceed through appropriate cell, primary-cell or organoid, animal, and, where relevant, aged-animal models before consideration of clinical development. Brain-targeted or intestinal-targeted delivery systems may be explored as preclinical strategies, but their efficacy, biodistribution, and safety require experimental validation.

#### 5.2.3 Research system and technology optimization for redox studies in aging context

For JWA and aging research, it is necessary to construct aging-specific research models and optimize detection technologies to simulate the *in vivo* aging microenvironment and accurately detect JWA’s expression and function in aging cells and tissues. For model construction and application: Construct JWA conditional knockout, tissue-specific overexpression and knock-in animal models in aged mice, and age-stratified PDX models representing relevant patient age groups and humanized aging mouse models to simulate the *in vivo* aging redox microenvironment, verifying JWA’s functions in tumor metastasis, neurodegeneration, intestinal homeostasis and other aging-related physiological and pathological processes (Wang et al., 2018; Li et al., 2022); establish a cell model library covering multiple aging stages, disease subtypes and redox contexts (e.g., primary human aging fibroblasts, neural stem cells, intestinal stem cells) to investigate JWA functional specificity in different aging states (Chen H. et al., 2007; Siddique et al., 2023). Construct human gut microbiota-aged mouse chimeric models to explore the crosstalk between JWA, gut microbiota and redox balance in the aging process.

For detection technology and research method innovation: Develop high-sensitivity and high-specificity JWA expression detection technologies (e.g., digital PCR, single-molecule immunoassays) to achieve precise quantification of JWA and related redox molecules in body-fluid and tissue samples collected across relevant age groups (Butchbach et al., 2002; Butchbach et al., 2003); combine single-cell sequencing and spatial transcriptomics to analyze JWA’s cell-specific expression patterns and redox-context-dependent functional heterogeneity in the aging tissue microenvironment (e.g., aging brain, intestinal tissue, tumor microenvironment) (Wang et al., 2018; Siddique et al., 2023); utilize bioinformatics to construct a panoramic JWA redox signaling pathway network in the aging process, and explore potential interacting molecules and target nodes (Hu et al., 2025); develop non-invasive detection methods for JWA expression (e.g., circulating exosome JWA detection) for the future biomarker evaluation and longitudinal monitoring of age-associated diseases.

#### 5.2.4 Expansion of redox-related functional research domains and combination therapy exploration for aging

For JWA research, it is necessary to expand the research scope to various age-related diseases, explore the common regulatory mechanisms of JWA in oxidative stress and aging, and develop personalized combination intervention strategies. For cross-disease functional research: Expand JWA functional studies to redox-sensitive age-related diseases, metabolic diseases, and environment-related diseases (Cao et al., 2007; Chen R. et al., 2007; Yamamoto and Sakisaka, 2025b); further verify JWA’s broad-spectrum neuroprotective potential in clearing abnormal protein aggregates via stress-associated reticulophagy in other neurodegenerative diseases, and explore its regulatory role in cognitive decline and memory impairment in aging or neurodegenerative disease models (Wang et al., 2018; Wang W. et al., 2020); investigate JWA’s role in regulating gut microbiota-intestinal epithelial redox homeostasis in the aging process, and clarify its impact on age-related intestinal barrier damage and systemic inflammation (Li et al., 2022; Zhou et al., 2022); explore the regulatory role of JWA in immune senescence and age-related inflammatory responses (inflammaging), and clarify its mechanism of action in regulating the redox regulation of immune cells during aging.

For combination therapy strategy exploration: Explore synergies between JWA-targeted redox therapy and existing treatments in the elderly populationexisting treatments in age-associated disease context includes evaluating the combined efficacy of JWA-modulating strategies in gastric cancer models representing relevant age-associated contexts; verifying synergies of JWA modulators with chemotherapeutic drugs and immune checkpoint inhibitors in relevant tumor models, with age-dependent effects requiring future evaluation, and identifying optimal combination regimens to reduce toxicity and adverse effects (Liu et al., 2012; Ye et al., 2013; Wu X. et al., 2014; Xu et al., 2018; Chen et al., 2020). Explore combined application of JWA with other DNA repair pathway inhibitors (e.g., poly(ADP-ribose) polymerase (PARP) inhibitors) and neuroprotective agents (e.g., neurotrophic factors) to provide more efficient treatment combinations for age-redox dysregulation-related diseases (Sha et al., 2016; Wang X. et al., 2020). In addition, strengthen the research on the long-term safety, pharmacokinetics, pharmacodynamics, and age-dependent toxicity of JWA-targeted strategies, and provide a scientific basis for its clinical application.

## 6 Conclusion

JWA is best understood not as a universal regulator of redox homeostasis or a direct ROS sensor, but as a context-dependent stress-responsive regulatory protein that intersects with redox-associated cellular processes. Its functional consequences depend on cellular identity, basal redox state, stress intensity, DNA-repair demand, proteostatic burden, and disease context.

This context dependence provides a framework for interpreting apparently divergent observations across the JWA literature. For example, JWA can support XRCC1 stability and oxidative DNA repair in normal cells, whereas distinct regulatory states in therapy-resistant cancer cells can produce an opposite effect on XRCC1 and chemotherapy response. Similarly, neuronal studies indicate that JWA can protect dopaminergic neurons from ferroptotic injury while JWA deficiency enhances hippocampal neurogenesis and synaptic plasticity in another experimental context. These findings should therefore not be reduced to a single universal direction of JWA activity.

Within the aging framework, current evidence supports an association between JWA-related stress regulation and selected age-associated disease processes, but direct evidence establishing JWA as a universal regulator of biological aging remains limited. The major unresolved question is whether age-dependent changes in JWA expression or activity are causal determinants of tissue dysfunction or instead reflect downstream responses to altered cellular stress, DNA-repair demand, and proteostatic burden. Addressing this question will require aging-specific models, longitudinal studies, tissue-resolved analyses, and age-stratified human datasets.

From a translational perspective, JWA-related expression patterns, genetic variants, and pharmacological modulators remain candidate or experimental opportunities rather than clinically validated tools. Future studies should prioritize mechanism-specific validation, biomarker standardization, pharmacological characterization, and disease-specific preclinical evaluation before clinical development can be considered. Thus, the principal value of JWA research at present lies in defining when and how this context-dependent stress-response network influences redox-associated tissue adaptation, rather than assuming a universal therapeutic direction. The central challenge is therefore not simply to determine whether JWA is protective or detrimental, but to define the biological conditions under which JWA-associated stress regulation produces distinct functional outcomes.

## Glossary

AKT: Protein Kinase B
Akt-CREB: Protein Kinase B/cAMP Response Element Binding Protein
AMPK: Adenosine 5′-Monophosphate-Activated Protein Kinase
**As**_**2**_ **O**_**3**_: Arsenic Trioxide
ATG12: Autophagy-Related Gene 12
ATG5: Autophagy-Related Gene 5
ATRA: All-Trans Retinoic Acid
Bax: BCL2-Associated X Protein
Bcl-2: B-Cell Lymphoma 2
BER: Base Excision Repair
BRCA1: Breast Cancer Susceptibility Gene 1
C/EBP: CCAAT/Enhancer Binding Protein
CALCOCO1: Calcium Binding and Coiled-Coil Domain 1
CDK6: Cyclin-Dependent Kinase 6
CHRNA5: Cholinergic Receptor Nicotinic Alpha 5 Subunit
CK2: Casein Kinase 2
CTBP1: C-Terminal Binding Protein 1
CXCR4: C-X-C Chemokine Receptor 4
E2F1: E2F Transcription Factor 1
EAAC1: Excitatory Amino Acid Carrier 1
EAAT3: Excitatory Amino Acid Transporter 3
EGFR: Epidermal Growth Factor Receptor
ER: Endoplasmic Reticulum
ERK: Extracellular Signal-Regulated Kinase
FAK: Focal Adhesion Kinase
FAM134B: Family with Sequence Similarity 134, Member B
FBXW7: F-box and WD Repeat Domain Containing
FTH1: Ferritin Heavy Chain 1
GLT-1: Glutamate Transporter 1
GSH: Glutathione
HRE: Heat Shock Response Element
HSF1: Heat Shock Factor 1
IHC: Immunohistochemistry
ILK: Integrin-Linked Kinase
ING4: Inhibitor of Growth Family Member 4
JAC1: JWA Gene Activating Compound 1
JAC4: JWA Gene Activating Compound 4
JNK: c-Jun N-Terminal Kinase
K158: Lysine 158
K716: Lysine 716
LC3: Autophagosome Marker
MAP: Microtubule-Associated Protein
MAPK: Mitogen-Activated Protein Kinase
MDM2: Murine Double Minute 2
MEK: Mitogen-Activated Protein Kinase
MMP-2: Matrix Metalloproteinase-2
MPTP: 1-Methyl-4-Phenyl-1,2,3,6-Tetrahydropyridine
mTOR: Mammalian Target of Rapamycin
NCOA4: Nuclear Receptor Coactivator 4
NEDD4L: Neural Precursor Cell Expressed Developmentally Downregulated 4-Like
NFI: Nuclear Factor I
NF-κB: Nuclear Factor-kappa B
NOTCH1: Notch Homolog 1
Nrf2: Nuclear Factor Erythroid 2-Related Factor 2
OSAHS: Obstructive Sleep Apnea-Hypopnea Syndrome
p21: Cyclin-Dependent Kinase Inhibitor 1A
p38: p38 mitogen-activated protein kinase
p53: Tumor Protein p53
PARP: Poly ADP-Ribose Polymerase
PCR-RFLP: Polymerase Chain Reaction-Restriction Fragment Length Polymorphism
PDX: Patient-Derived Xenograft
PI3K: Phosphatidylinositol 3-Kinase
PKA: cAMP-Dependent Protein Kinase
PKC: Protein Kinase C
PL-OOH: Lipid-Peroxidation Products.
PPARγ: Peroxisome Proliferator-Activated Receptor γ
PRA1: Prenylated Rab Acceptor 1
Pt(IV): Platinum(IV)
RARE: Retinoic Acid Response Element
RNF185: Ring Finger Protein 185
ROS: Reactive Oxygen Species
RRID: Resource Identification Initiative
RT-qPCR: Reverse Transcription Quantitative Polymerase Chain Reaction
SDR: Serine-Aspartic acid-Arginine
siRNA: Small Interfering RNA
SLR: Serine-Leucine-Arginine
Sp1: Specificity Protein 1
SRE: Stress Response Element
STAT3: Signal Transducer and Activator of Transcription 3
STAT5: Signal Transducer and Activator of Transcription 5
TH: Tyrosine Hydroxylase
TIMP2: TIMP Metallopeptidase Inhibitor 2
TKI: Tyrosine Kinase Inhibitor
TMDs: Transmembrane Domains
TNBC: Triple Negative Breast Cancer
TPA: 12-O-Tetradecanoylphorbol-13-Acetate
TRE: TPA Response Element
Ub: Ubiquitin
VEGF: Vascular Endothelial Growth Factor
XRCC1: X-Ray Repair Cross Complementing 1
YY1: Yin Yang 1
